# Bonding durability and remineralizing efficiency of orthodontic adhesive containing titanium tetrafluoride: an invitro study

**DOI:** 10.1186/s12903-023-03063-2

**Published:** 2023-05-30

**Authors:** Omnia Mahmoud Khalifa, Manal Farouk Badawi, Tarek Ahmed Soliman

**Affiliations:** 1 Dental Biomaterials Department, Faculty of Dentistry, Mansoura University, Mansoura, Egypt; 2 Prosthetic Dentistry Department, Faculty of Dentistry, New Mansoura University, Mansoura, Egypt

**Keywords:** Bonding, Cariogenic challenge, Orthodontic adhesive, Titanium tetrafluoride

## Abstract

**Background:**

Titanium tetrafluoride has been shown to protect tooth enamel from demineralization. This study investigated the effect of incorporating different concentrations of TiF4 (1, 2 and 3 Wt.%) into an orthodontic primer on the shear bond strength of orthodontic brackets and the enamel microhardness after cariogenic challenges.

**Methods:**

Three different TiF4 concentrations (1, 2 and 3 Wt.%) were prepared and added to the etch and rinse orthodontic primer. Ninety freshly extracted premolars were randomly divided into five groups according to the experimental primers and ageing conditions: TF0, TF0C, TF1C, TF2C, and TF3C. The TF0C group had no TiF4 in the primer, while TF1C, TF2C, and TF3C had 1, 2 and 3 Wt.% TiF4 in the primer, respectively. In the TF0 group, specimens were immersed in deionized water for 24 h as a control group, while all other groups were immersed in a demineralizing solution for 28 days. Each of the five groups was divided into two subgroups: The first group was subjected to shear bond strength and adhesive remnant index testing (*N* = 50 teeth, 10/group), while the second group was subjected to enamel surface microhardness testing (*N* = 25 teeth, 50 tooth halves, 10 tooth halves/group). Fifteen teeth (*N* = 15 teeth, *n* = 3/group) representing the five groups were subjected to SEM and microelemental analysis (EDX). SBS, ARI, microhardness, and Ca/P ratio were measured, and the data were analyzed using ANOVA and Tukey's tests.

**Results:**

The TF2C group had the highest SBS value (9.93 ± 1.23), while the TF0C (5.24 ± 0.65) and TF3C (5.13 ± 0.55) had the lowest SBS values. The enamel microhardness in the TF0C group was significantly reduced (*p* < .001). Enamel microhardness values were significantly (*p* < .001) higher in groups TF1C, TF2C, and TF3C than in TF0C. The highest Ca/P ratio was significantly recorded for the TF2C group (2.65 ± 0.02).

**Conclusions:**

Incorporation of 1 and 2 Wt.% TiF4 into the orthodontic primers showed adequate bond strength and better remineralization effect. However, 1 Wt.% TiF4 showed lower ARI values than 2 Wt.% TiF4.

## Introduction

The bond between orthodontic brackets and enamel must be maintained during orthodontic therapy to ensure effective force application [1, 2]. One of the drawbacks of orthodontic treatment is the demineralization of the tooth enamel in the region around and under the bonded orthodontic brackets. The smoothness and hardness of the enamel surface, as well as the bond strength of the bracket to the enamel, may be affected by this demineralization [3–5].

Continuous attempts have been made to treat peri-bracket white spot lesions by preventing enamel demineralization or promoting enamel remineralization [6, 7]. Topical fluoride has been shown to minimize enamel demineralization around brackets. Conversely, fluoride treatment prior to bracket insertion makes the enamel resistant to phosphoric acid etching, which in turn reduces the effectiveness of the bond by causing early degradation of the bond [8–10].

Titanium tetrafluoride (TiF4) has been shown to provide better protection against enamel demineralization. Due to the unique interaction of TiF4 with tooth structure, enamel absorbs fluoride faster and better, which also leads to the deposition of CaF2 and the formation of an acid-resistant titanium dioxide (TiO2) layer [11, 12]. TiF4 is a promising anticaries agent; however, due to its strong interactions with all fluids and unstable pH, it cannot be used alone for treatment. Technically, the addition of TiF4 to the primer or bonding agent would facilitate its use [13].

Basting et al. [14] investigated the effects of adding TiF4 (2.5 Wt.%) to the primer or bond of a self-etching adhesive system on the long-term dentin bond strength and the physicomechanical properties of TiF4-containing adhesives. They concluded that TiF4-containing adhesives did not change the μTBS or failure mode over time but did affect the flexural strength and degree of conversion, especially when TiF4 was added to the bond. They also proposed further research to investigate the effects of adding TiF4 to a self-etching primer on its mechanical and physical properties and whether this approach would lead to biomimetic mineralization.

Since the acidic environment surrounding orthodontic brackets promotes demineralization and hinders the process of remineralization, and due to the ongoing controversy about TiF4 as an enamel surface pretreatment, the aim of this study was to investigate the effect of incorporating different concentrations of TiF4 (1, 2 and 3 Wt.%) into an orthodontic primer on the shear bond strength of orthodontic brackets and the enamel microhardness after cariogenic challenges. The null hypotheses were that the different TiF4 concentrations in the primer had no statistically significant effect on (1) enamel shear bond strength and (2) enamel microhardness after cariogenic challenge.

## Materials and methods

### Characterization of the experimental primer containing titanium tetrafluoride

**Fig. 1:**
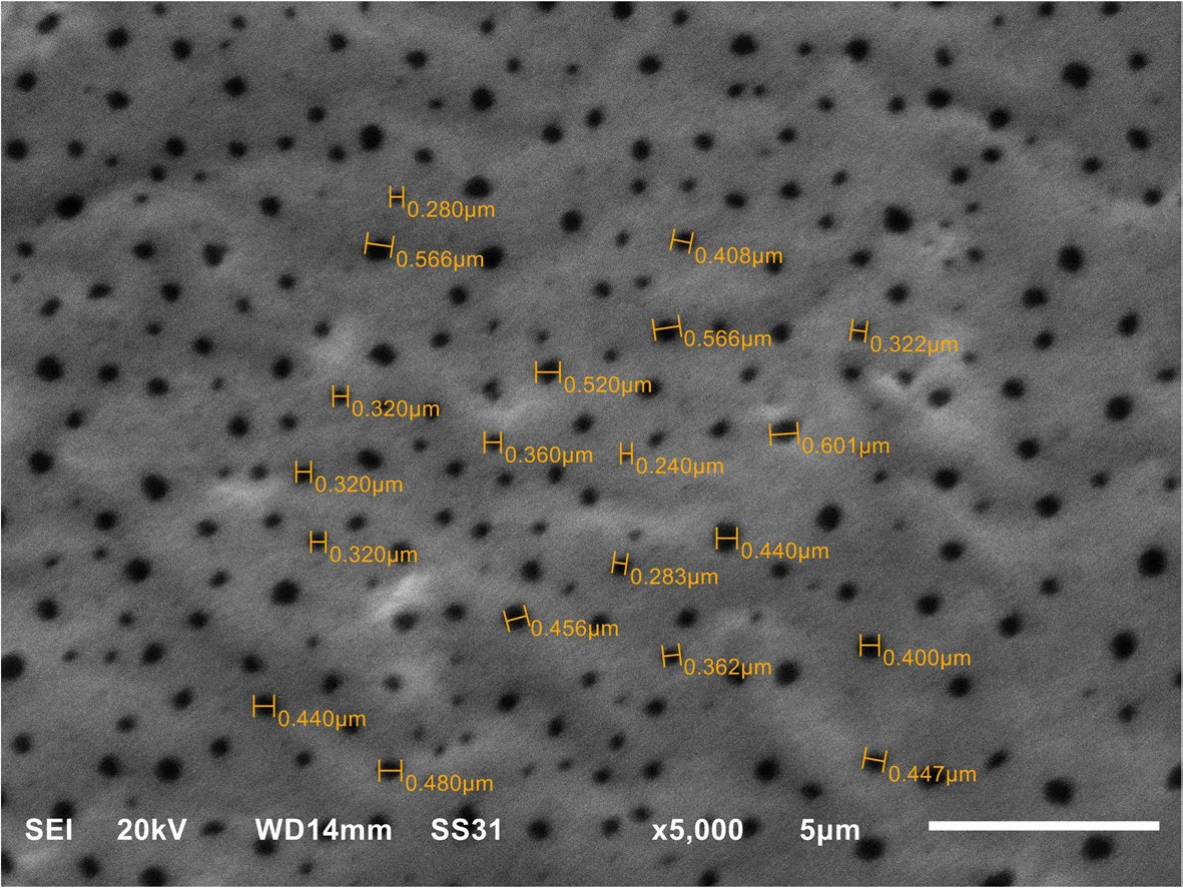
SEM of TiF4 distribution within the primer (5.000-X)

Commercial TiF4 crystals (Aldrich Chemical Company, Milwaukee, WI, USA) were ground to powder using a mortar and pestle, weighed, and added to the primer to achieve three different concentrations: 1, 2 and 3 Wt.%. With constant stirring with a sonicator, these concentrations were added to the orthodontic etch and rinse primer U Bond™ (VERICOM Co., Ltd., lot number 0p190100) and mixed vigorously to ensure homogeneous distribution to formulate three different concentrations: TF 1 (10 mg TiF4 mixed with 1 mL primer), TF 2 (20 mg TiF4 mixed with 1 mL primer), and TF 3 (30 mg TiF4 mixed with 1 mL primer). In addition, TF0 (without the TiF4 addition) was used as a control group. All experimental primers were stored in tightly sealed vials, protected from light and humidity. The distribution of 3 Wt.% TiF4 in the primer was evaluated by scanning electron microscopy analysis (Jeol-JSM-6510, Tokyo, Japan) at an original magnification of 5000-x. The particle size was calculated using an analysis tool in Image J software and was in the range of 28–60 nm (Fig. 1).

The degree of conversion of the TiF4-containing primers was evaluated using Fourier Transformation Infrared Spectroscopy (FTIR) with an attenuated total reflectance (ATR) technique [4]. To obtain reference values for aromatic and aliphatic band rings, droplets of unpolymerized primer were either incorporated with TiF4 or left unincorporated. The experimental primers were freshly manipulated and applied to the device's crystal surface. A standard distance of 1 mm was maintained between the light device's tip and the light-cured specimens. After 10 s of light curing, the degree of conversion was determined (five repetitions/group). As an internal standard, the degree of conversion was estimated using polymerized and unpolymerized specimens based on the ratio between the carbon aliphatic and aromatic double bonds. The degree of conversion was calculated by the following equation:

$$DC \left(\%\right) =\left(100-\left(\frac{\frac{1638 cm-1}{1608 cm-1}polymerized}{\frac{1638 cm-}{1608 cm-1}unpolymerized}\right)\right)\times 100$$

### Specimen selection

Ninety freshly extracted premolars (*N* = 90) with intact buccal enamel were obtained from orthodontic patients whose treatment plans required extractions. They were preserved in thymol (0.1%, pH 7.0) for 24 h to inhibit bacterial growth. Premolars were selected based on integrity, absence of fractures and cracks, and demineralization of enamel. They were cleaned with a pumice slurry at low speed in a rubber prophy cup and then stored in distilled water at room temperature, which was changed regularly to prevent dehydration.

### Study design and specimens’ grouping

**Fig. 2:**
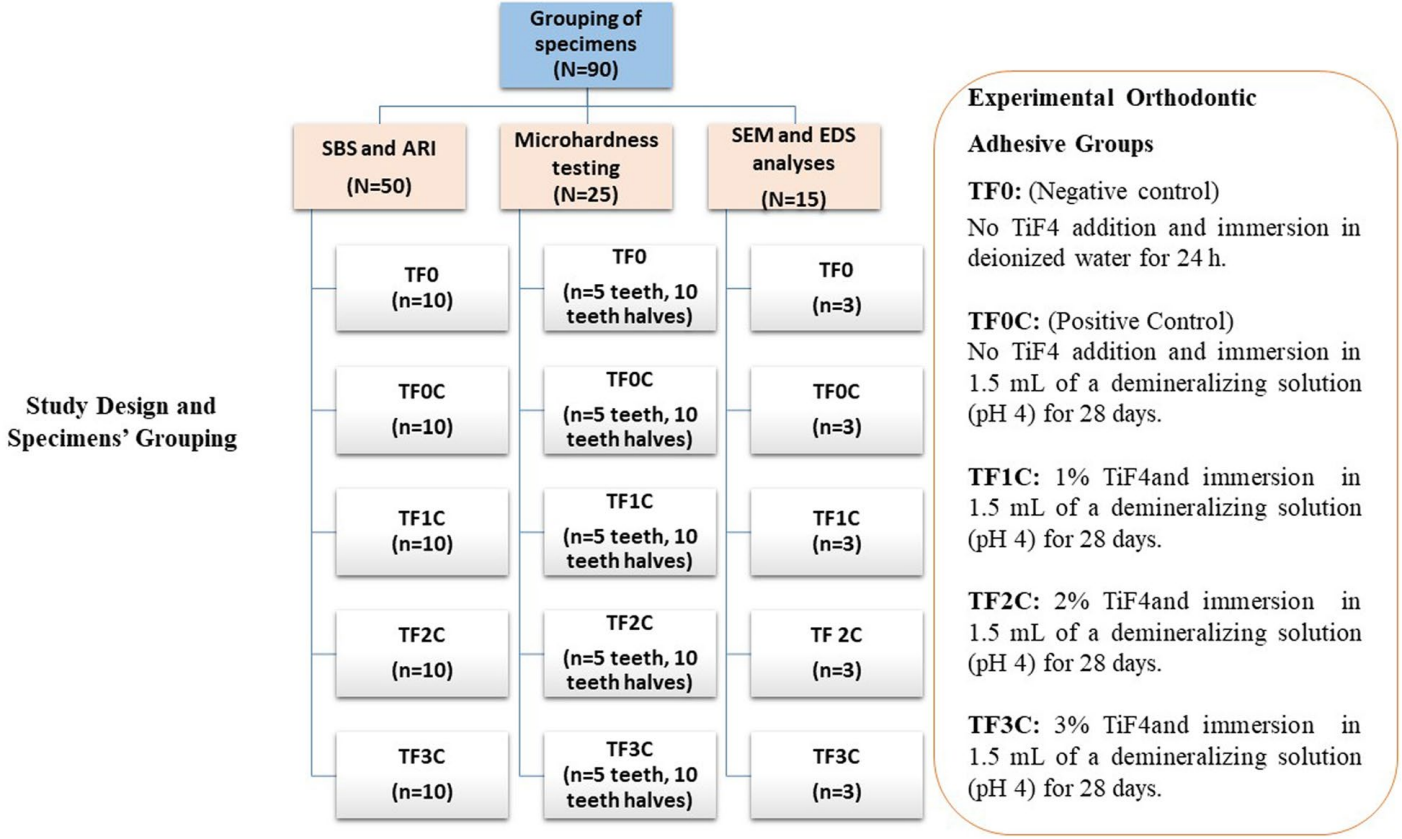
Study design and specimens’ grouping

Premolars were randomly divided into five groups (*n* = 15), which were subjected to five experimental conditions (Fig. 2): TF0 (no TiF4); specimens immersed in deionized water for 24 h; TF0C (no TiF4); specimens immersed in a demineralization solution for 28 days; TF1C, TF2C, and TF3C (primers contained 1, 2, and 3 Wt.% TiF4, respectively) and were then immersed in a demineralization solution for 28 days [14–16]. The demineralization solution consisted of "3.0 mmol/L CaCl2, 1.8 mmol/L KH2PO4, 0.1 mol/L lactic acid, and 1% carboxymethylcellulose," and the pH was adjusted to 4 with KOH [15, 17]. The solution was changed daily to ensure that the pH was constant at 4. Each group consisted of ten specimens based on power analysis, giving a power of 0.95 at a significance level of 0.05 [16]. Each of the five groups was divided into three subgroups; the first group was subjected to shear bond strength and adhesive remnant index testing (*N* = 50 teeth, 10/group), and the second one was subjected to enamel surface microhardness testing (*N* = 25 teeth, 50 tooth halves, 10 tooth halves/group). Additionally, fifteen teeth (*N* = 15 teeth, *n* = 3/group) were subjected to SEM and microelemental analysis (EDX).

### Preparation for enamel-bracket bonding

The buccal enamel surface of each premolar was etched with 37% phosphoric acid (Scotch Bond, 3 M ESPE, St. Paul, MN, USA) for 30 s, rinsed with water for 30 s, and then properly dried with oil- and moisture-free air. As prescribed by the manufacturer, experimental primers were applied to the buccal surface of the premolars. A thin, uniform layer of the experimental orthodontic primers was applied to the etched surface. The adhesive was applied to the base of the brackets before insertion. The orthodontic metal brackets (Geousis Mini Roth, ORMCO, CA, USA; lot number: 120114) were then lightly placed on the tooth surface and firmly pressed into their final position. A half-kg customized metallic tool was used to apply standard, uniform pressure to the top of the brackets during the bonding process, resulting in uniform adhesive thickness. An explorer was used to remove the excess adhesive. The entire adhesive resin was polymerized for 12 s in two directions (6 s each) using the Ortholux Luminous Curing Light (3 M Unitek; Monrovia, California, USA; light output: 1600 mW/cm2) [2]. Subsequently, the bonded premolars were stored in distilled water at 37 °C for 24 h to allow proper polymerization of the bonding process.

### Bracket Shear Bond Strength (SBS) testing and Adhesive Remnant Index (ARI)

The SBS test was performed using a universal testing machine. In order for the bonded bracket base to be parallel to the direction of the shear force, the test specimens had to be fixed in the lower jaw of the machine. A compressive force was applied to the specimens at a crosshead speed of 0.5 mm/min [2, 18]. The base of the bracket was precisely aligned with the stainless-steel rod with a mono-beveled chisel that was attached to the upper moving shelf of the testing machine. The fracture load (F) in Newtons was divided by the surface area (A) in mm^2^ to determine the SBS in megapascals (MPa). By measuring the length and width with a digital caliper (Mitutoyo Corporation, Tokyo, Japan) and then calculating the area, the bonding area of the bracket was determined [2]. After debonding, the remaining adhesive on the enamel surface was assessed by examining the fractured specimen under a 20 × optical stereo microscope (Olympus SZ61, Tokyo, Japan). The evaluation was conducted based on the following standards [19]: 0 = no adhesive remaining on enamel; 1 = less than half of the adhesive remaining on enamel; 2 = more than half of the adhesive remaining on enamel; 3 = all the adhesive remaining on enamel. The ARI scores will be used to determine bond failure sites between the enamel, adhesive resin, and bracket base.

### Microhardness testing

The degree of enamel demineralization was indirectly determined by microhardness testing. The bracket-buccal bonded interface was positioned flat, in contact with the bottom of a tube containing the demineralizing solution, and fully submerged in 1.5 mL of the solution (pH 4).The specimens were then cut sagittally into mesial and distal halves at the center of the bracket using a low-speed water-cooled diamond saw. The cut face of the sectioned specimens was left exposed as they were embedded in acrylic resin blocks (Paladur, Heraeus-Kulzer, Hanau, Germany).Wet silicon carbide papers (Microcut ™, Buehler, Lake Bluff, USA) with different grades (600, 800, and 1200 grit) were used to polish the cut surfaces of the resin-embedded specimens. Diamond cream and a 1 μm polishing-cloth disc were used for the finishing polish. A hardness tester with a Vickers diamond indenter was used to measure the microhardness, with a load of 50 g and a dwell time of 15 s. Under the bracket, 30 μm from the exterior enamel surface, indentations were produced on the buccal surface [16]. Three readings were averaged to get one from each specimen.

### Scanning Electron Microscopy (SEM) and energy-dispersive x-ray Spectroscopy (EDS) analyses

Three additional specimens from each group were prepared as in surface microhardness testing. The cut polished specimens were cleaned for two minutes in an ultrasonic bath with 96% ethanol, and then they were dried by air.

Three additional specimens from each group were prepared in the same manner as in the surface microhardness test. The cut and polished specimens were cleaned in an ultrasonic bath with 96% ethanol for two minutes and then air dried. The specimens were mounted on metallic stubs, coated with gold sputter, and then examined under a SEM (Jeol-JSM-6510, Tokyo, Japan) at an original magnification of 600 × to analyze the enamel surface and bracket/adhesive/enamel junction. In addition, the specimens were examined at EDS using energy dispersive X-ray microanalysis (EDX) to measure the weight percentages of calcium and phosphorus ions under the bracket in close proximity to the bracket/adhesive/enamel junction interface. Three measured values of ion weight percentages were averaged to obtain one value per specimen [20, 21].

### Statistical analysis

The normality and equal variance assumptions were fulfilled according to the Shapiro–Wilk test (*p ˃ 0.05)* and Levene's test. A one-way ANOVA was conducted to analyze the SBS, hardness, and *Ca/P* ratio data regarding the five experimental conditions. Tukey's significant difference tests were used for post-hoc comparisons. The Chi-square (χ^2^*)* test was used to determine significant differences in the ARI scores among the five experimental groups. The level of significance was set at 5% for all statistical tests.

## Results

TiF4 nanoparticles were identified in scanning electron microscopy. The particle size observed in SEM was 28–60 nm. One-way ANOVA showed that there were no statistically significant differences in the degree of conversion (*p* = 0.981) among TF0, TF1, TF2,TF3 groups (81.76 ± 4.9, 84 ± 2.8, 82.4 ± 3.2, 83.6 ± 2.2) respectively.

The means ± SD of SBS values (MPa) for all groups are shown in Table 2. A one-way ANOVA test showed significant differences among the five experimental groups (*p* < 0.001, F = 16.09). The Tukey test for significant differences showed that the highest SBS value was obtained for the TF2C group (9.93 ± 1.23), while the lowest SBS value was obtained for TF0C (5.24 ± 0.65) and TF3C (5.13 ± 0.55). The TF2C group recorded a significantly higher SBS value (*p* < 0.001) (9.93 ± 1.23) compared to all other groups except the TF0 group (8.02 ± 1.55) (*p* = 0.09). The five experimental conditions significantly affect the ARI values according to the Chi-square (χ2) test and Monte Carlo test as a correction for Chi-square (*p* < 0.001, χ2MC = 31.04). A closer look at the data in Table 1, TF0, TF0C, and TF3C showed a high incidence of scores 0 (50%, 70%, and 80%, respectively). However, TF1C recorded a high incidence of scores 1 and 2 (50% and 20%, respectively). TF2C showed a high incidence of scores 2 and 3 (30% and 60%, respectively).

**Table 1:** Shear Bond Strength (SBS) and Adhesive Remanent index (ARI) scores for the different experimental groups

Groups	SBS	ARI Scores			
		0	1	2	3
TF0	8.02 ± 1.55 ^ba^	5	2	2	1
TF0C	5.24 ± 0.65 ^d^	7	3	0	0
TF1C	7.79 ± 1.75 ^cb^	2	5	2	1
TF2C	9.93 ± 1.23 ^a^	0	1	3	6
TF3C	5.13 ± 0.55 ^ed^	8	2	0	0

One way ANOVA for SBS: *p* < .001, *d f* = 4, Mean Square = 24.95, F = 16.09

Mean values represented with different superscript lowercase letters (column) is significantly different according to Tukey’s significant different test (*P* < *.05)*

The means ± SD of Vickers hardness values for all groups are shown in Table 2. A one-way ANOVA test showed significant differences among the five experimental groups (*p* < 0.001, F = 34.46). The Tukey test showed that the highest hardness value was obtained for the TF3C group (377.40 ± 55.10), while the lowest value was obtained for the TF0C group (184.48 ± 32.93). As for the effects of demineralization, enamel microhardness was significantly reduced in the TF0C group (*p* < 0.001). In the TF1C, TF2C, and TF3C groups, the effect of different TiF4 concentrations significantly increased the enamel microhardness compared to the positive control group (TF0C) (*p* < 0.001). No significant differences were observed between TF1C and TF2C (*p* = 0.89). Enamel microhardness was not significantly higher in both TF1C and TF2C compared to the negative control group (TF0), (*p* = 0.89 and *p* = 0.61, respectively).

**Table 2:** Cross-sectional Microhardness and ions concentrations for the different experimental groups

Groups	Cross-Sectional Microhardness	Ions Concentrations (wt.%)		
		Ca	*P*	Ca/P ratio
TF0	306.90 ± 14.27 ^d^	37.99 ± 0.22 ^c^	17.75 ± 0.21 ^ba^	2.14 ± 0.03 ^c^
TF0C	184.48 ± 32.93 ^e^	22.43 ± 0.70 ^e^	13.24 ± 0.23 ^e^	1.69 ± 0.02 ^e^
TF1C	322.19 ± 45.72 cd	33.44 ± 0.59 ^d^	15.95 ± 0.19 ^d^	2.09 ± 0.04 ^dc^
TF2C	328.71 ± 32.63 ^bd^	45.01 ± 0.18 ^b^	16.99 ± 0.19 ^ca^	2.65 ± 0.02 ^ab^
TF3C	377.40 ± 55.10 ^a^	47.14 ± 0.55 ^a^	17.93 ± 0.41 ^a^	2.63 ± 0.09 ^b^

One way ANOVA for Ca ions: *p* < .001, *df* = 4, Mean Square = 294.58, F = 1197.55

One way ANOVA for P ions: *p* < .001, *df* = 4, Mean Square = 11.04, F = 163.87

One way ANOVA for Ca/P ratio: *p* < .001, *df* = 4, Mean Square = .485, F = 195.66

^*^ Mean values represented with different superscript lowercase letters (column) is significantly different according to Tukey’s significant different test (*P* < *.05)*

The results of the statistical analysis of the Ca/P ratio are shown in Table 2. The Tukey test showed that the Ca/P ratio was significantly highest in the TF2C group (2.65 ± 0.02), whereas the lowest value was recorded for the TF0C (1.69 ± 0.02). The Ca/P ratio was significantly reduced in the TF0C group (*p* < 0.001). In the TF1C, TF2C, and TF3C groups, the Ca/P ratio was significantly higher than in the positive control (TF0C) group (*p* < 0.001). In both TF2C and TF3C, the Ca/P ratio was significantly higher than in the negative control (TF0) group (*p* < 0.001). No significant difference was found between the TF1C and negative control (TF0) group (*p* = 0.82). In addition, EDS showed the presence of titanium ions in TF1C, TF2C, and TF3C (1.86, 1.90, and 2.34 Wt.% respectively).

**Fig. 3:**
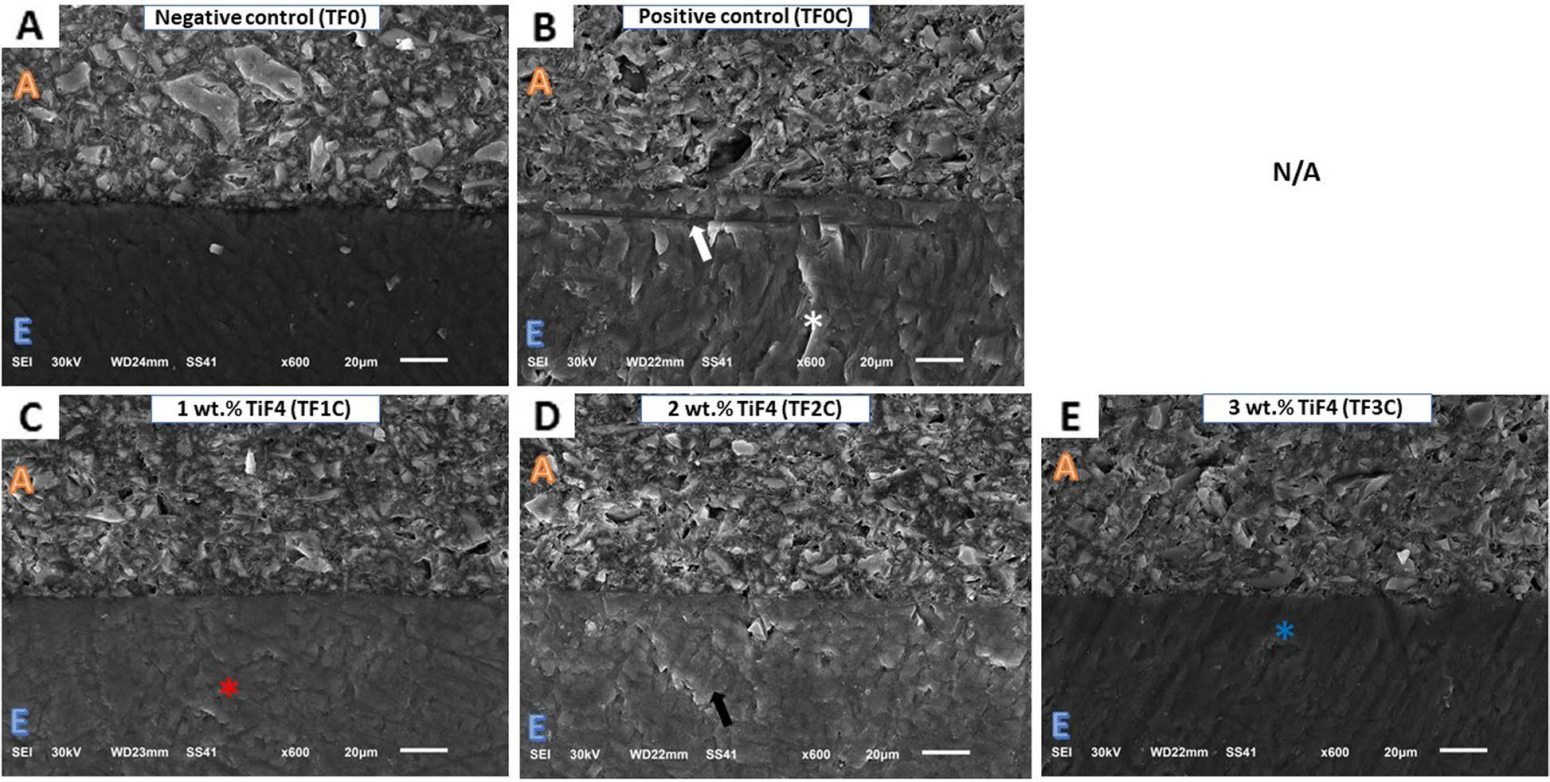
SEM micrographs (600x) of the different experimental groups: adhesive (**A**), enamel surface (**E**), hybrid zone (white arrow), irregular enamel surface with apparent cracks (white asterisk), homogenous enamel surface with crystal recovery (red asterisk), restored enamel crystal on the demineralized enamel (black arrow), irregular enamel surface with grooves and pits (blue asterisk)

SEM images at 600 × magnification showed different morphological features of the enamel surface for each group (Fig. 3). The TF0 group (Fig. 3A) showed the normal common component of the intact crystalline enamel structure. In the TF0C group (Fig. 3B), the integrity of the enamel prisms was severely compromised and exhibited an irregular surface with obvious cracks in both the enamel surface (white asterisk) and the hybrid zone (white arrows), which could affect the bonding integrity with the orthodontic adhesive. In group TF1C, an inhomogeneous enamel surface with the recovery of enamel prism crystals (red asterisk) and a discontinuous fish scale texture were observed (Fig. 3C).

Group TF2C showed a rough enamel surface and recovered enamel crystals (black arrow) on the demineralized enamel surface (Fig. 3D). Group TF3C showed an intact, non-homogeneous, irregular enamel surface with grooves and pits on the enamel surface (blue asterisk) (Fig. 3E).

## Discussion

Demineralization around orthodontic brackets remains a clinical concern for orthodontists during and after orthodontic treatment, especially when patients do not follow oral hygiene instructions. Therefore, the optimal orthodontic adhesive should maintain the integrity of the enamel surface during bracket removal while providing adequate enamel bond strength to prevent bracket detachment during tooth movement [22, 23].

Titanium tetrafluoride is a fluoridated compound with low acid solubility that can significantly protect tooth enamel from demineralization by depositing CaF2 and forming an acid-resistant titanium dioxide (TiO2) layer on the enamel [24]. The use of TiF4 has been proposed as a strategy to minimize the degradation of the hybrid layer, reduce the incidence of secondary caries, and ensure adequate bond strength of orthodontic brackets. Although TiF4 solution is a promising anticaries compound, it is unstable and has a low pH [24, 25]. Due to the ongoing debate about TiF4 as an enamel pretreatment and the lack of studies with conventional bonding systems, the incorporation of TiF4 into the three-step etch-and-rinse primer may be critical to the longevity of the hybrid layer and the potential inhibition of secondary carious lesions. Furthermore, titanium tetrafluoride (TiF4) was used in most studies at concentrations ranging from 0.1 to 4% [22, 23]. This raises the question of which TiF4 concentration is suitable for clinical use to reduce enamel demineralization under orthodontic brackets. Therefore, the aim of the present study was to investigate the effect of incorporation of different concentrations of TiF4 (1, 2, and 3 Wt.%) in the three-step etch-and-rinse primer on the shear bond strength of orthodontic brackets and enamel microhardness after cariogenic exposure.

Furthermore, titanium tetrafluoride (TiF4) was used in concentrations between 0.1 and 4% in most studies [22, 23]. This raises the question of which TiF4 concentration is suitable for clinical use to reduce enamel demineralization under orthodontic brackets. The present study therefore aimed to investigate the influence of incorporating different concentrations of TiF4 (1, 2 and 3 Wt.%) into the three-step etch and rinse primer on the shear bond strength of orthodontic brackets and the microhardness of enamel after cariogenic exposure.

The shear test is the most common laboratory technique for evaluating the shear bond strength of brackets [2, 18]. The shear wedge blade used in this study was a stainless-steel rod with a mono-beveled chisel configuration. The crosshead speed was set between 0.5 and 1.00 mm/min because it allows more uniform stress distribution at the bonded interface and is useful, fast, and less sensitive to handling during setting [26]. The shear wedge was precisely positioned on the bracket base to avoid further rotational stresses, although the debonding forces occurring in vivo are more likely to be applied to the bracket wings [2].

Metal brackets should ideally have a shear bond strength of 7–9 MPa to the enamel to ensure adequate adhesion and facilitate bracket removal after orthodontic treatment [27]. The ARI score was used to measure the residual adhesive on the enamel surface to determine the location of deboned interface failure during shear bond strength testing. The lower the ARI value, the easier the cleaning process after debonding [28]. In the current study, a demineralization solution was used to mimic the cumulative effects of clinical use over a more realistic period of time [16]. Previous studies have shown that dental biofilms produce acids with a pH of 4. Therefore, an in vitro model for the rapid development of white spot lesions was prepared using a demineralization solution with a pH of 4 [29–31].

Based on the SBS results, the incorporation of TiF4 into the orthodontic primer has a significant effect on SBS; therefore, the first null hypothesis must be rejected. Enamel shear bond strength was significantly lower in the TF0C group (the positive control) than in the TF0 group (the negative control). This suggests that the acidic environment significantly compromised the bonding interface between the TFC0 adhesive and enamel, resulting in dissolution and loss of minerals (Ca and P ions), thus reducing bond strength [16, 22, 24]. This is supported by three factors: first, lower ARI values for the TFC0 group, indicating adhesive failure; second, the presence of an obvious crack in the hybrid zone, as shown in the SEM image (Fig. 3B), which could affect the integrity of the bond with the orthodontic adhesive; and third, lower Ca and P ions (22.43 ± 0.70 and 13.24 ± 0.23, respectively) in the TF0C group compared with the TF0 group (37.99 ± 0.22 and 17.75 ± 0.21, respectively), as shown in EDS.

The incorporation of TiF4 into the primer was suggested to simplify the hybridization sequence and to benefit from its use as an enamel biomodifier [14]. When TiF4 was included in the primer at different concentrations (1, 2, and 3 Wt.%), an increase in enamel bond strength above 7 MPa was observed in the TF1C and TF2C groups compared to the positive control group (TF0C), which could lead to a clinically acceptable application. The increase in bond strength could be a result of the addition of TiF4 as a filler additive to the primer [32]. In addition, TiF4 could contribute to the improvement of the mechanical properties of the hybrid layer by forming titanium oxides (the titanium ions in TF1C, TF2C, and TF3C were 1.86, 1.90, and 2.34 Wt.%, respectively), resulting in an acid-resistant, stable lining and higher resistance to tooth demineralization and thus higher bond strength [14, 20].

Rizvi et al. [33] investigated the effects of remineralization on bond strength and found that remineralizing agents can be clinically applied to the tooth surface to improve bond strength. These results are consistent with the ARI results in our study, as the ARI values were higher for TF1C (50% scores 1 and 20% scores 2) and TF2C (60% scores 3 and 30% scores 2).

In contrast, the TF3C group recorded a significantly lower SBS value (5.13 ± 0.55) than the clinically acceptable limit (7–9 MPa). This could be confirmed by the lower ARI value (80% score 0). Although 2 Wt.% TiF4 had clinically acceptable SBS, the reduction in SBS in the 3 Wt.% TiF4 group is difficult to explain but could be due to two reasons: first, the formation of a thick CaF2 precipitate layer, which could compromise the integrity of the bond and promote adhesive failure [23]; second, the higher viscosity of the primer after addition of TiF4 is due to the smaller particle size of TiF4 molecules (28–60 nm), which could hinder the penetration of the molecules into the hybrid zone and thus compromise the efficacy of the bond [10, 32].

The incorporation of TiF4 into the primer has a significant effect on the microhardness of the enamel; accordingly, the second null hypothesis has to be rejected. To analyze the microtopography of the underlying enamel surface, the brackets and adhesive must be removed, resulting in inaccurate data. Cross-sectional microhardness testing was used to estimate mineral loss by evaluating the area covered by the bracket, as it has a significant correlation (γ = 0.91) with the percentage of mineral loss [34–37]. Liu et al. [16] investigated the remineralization effect of an orthodontic adhesive containing MAE-DB on enamel hardness under the bracket at two depths (30 and 120 μm). At a penetration depth of 120 μm, there were no significant differences in microhardness values between the different surfaces, probably because this depth was not reached by acid etching. Therefore, a penetration depth of 30 μm was chosen for the present study.

The chemical composition of enamel strongly correlates with its hardness [10]. Previous studies have shown that demineralization leads to a loss of calcium and phosphate ions, which in turn affect the surface hardness of enamel. The microhardness values in the TF0C group decreased significantly in the current study, which was consistent with other results [12–14]. This showed that the resistance of enamel surfaces decreased as a result of exposure to demineralizing solution, which increases mineral loss and decreases the Ca/P ratio, thus decreasing the microhardness value. This was confirmed by the decrease in ion concentration, which is shown in Table 2. Hardness values increased for the TF1C, TF2C, and TF3C groups. Our results are in agreement with previous studies [10, 12, 38] that attribute the improved enamel microhardness to the ability of TIF4 to prevent demineralization by forming TiO2 in the presence of CaF2 and its remineralizing effect. The increase in ion concentration shown in Table 2 could also support this.

SEM–EDS has been used as a combined analytical technique to qualitatively examine the enamel surface and quantify the Ca/P ratio as an indicator of enamel health [39, 40]. In addition, EDS is a technique for chemical characterization and elemental analysis of materials in many studies [21, 41, 42]. Consequently, the Ca/P ratio and crystalline structural integrity are important indicators of enamel remineralization [41, 43]. Calcium and phosphorus are both present in hydroxyapatite, the main building block of tooth structure. A shift in the calcium/phosphate ratio implies a shift in the inorganic elements of hydroxyapatite. Previous studies have shown that demineralization leads to calcium and phosphate ion losses, thus reducing the Ca/P ratio. On the other hand, TiF4 can compensate for this loss, as shown by the results of the present study [12, 14, 16, 42].

An acceptable approach for a suitable orthodontic adhesive could be the incorporation of TiF4 into the primer. According to the results of this in vitro study, clinicians may be able to better understand the behavior of the enamel surface during de- and remineralization. This study has the limitation that it does not simulate the actual oral environment, as elements of the oral environment, including salivary components containing various minerals, lipids, carbohydrates, and proteins, as well as variations in pH, could affect bond strength. Nevertheless, further studies are needed to evaluate the pH and rheological properties of the experimental primers. In addition, a clinical performance evaluation is needed to make reliable recommendations for orthodontists.

## Conclusion

Within the limitations of this study, incorporation of 1 and 2 Wt.% TiF4 into the orthodontic primers showed adequate bond strength in relation to the clinically acceptable limit and better remineralizing potency. However, 1 Wt.% TiF4 showed lower ARI values than 2 Wt.%, implying easier cleaning after debonding of the orthodontic bracket.

## Data Availability

This article has all the data that were collected or analyzed during this study.

## References

[1] Reynolds IR (1975). A review of direct orthodontic bonding. Br J Orthod.

[2] Soliman TA, Ghorab S, Baeshen H (2022). Effect of surface treatments and flash-free adhesive on the shear bond strength of ceramic orthodontic brackets to CAD/CAM provisional materials. Clin Oral Investig.

[3] Julien KC, Buschang PH, Campbell PM (2013). Prevalence of white spot lesion formation during orthodontic treatment. Angle Orthod.

[4] Øgaard B, Rølla G (1988). Arends J Orthodontic appliances and enamel demineralization Part I lesion development. Am J Orthod Dentofac Orthoped.

[5] Richter AE, Arruda AO, Peters MC, Sohn W (2011). Incidence of caries lesions among patients treated with comprehensive orthodontics. Am J Orthod Dentofac Orthoped.

[6] Chin MY, Sandham A, Rumachik EN, Ruben JL, Huysmans MC (2009). Fluoride release and cariostatic potential of orthodontic adhesives with and without daily fluoride rinsing. Am J Orthod Dentofacial Orthop.

[7] Rogers S, Chadwick B, Treasure E (2010). Fluoride-containing orthodontic adhesives and decalcification in patients with fixed appliances: a systematic review. Am J Orthod Dentofacial Orthop.

[8] Øgaard B, Gjermo P, Rolla PG (1980). Plaque-inhibiting effect in orthodontic patients of a dentifrice containing stannous fluoride. Am J Orthod.

[9] Bridi EC, Amaral FLB, França FMG, Turssi CP, Basting RT (2013). Influence of dentin pretreatment with titanium tetrafluoride and self-etching adhesive systems on micro tensile bond strength. Am J Dent.

[10] Nassur C, Alexandria AK, Pomarico L, Sousa VP, Cabral LM, Maia LC (2013). Characterization of a new TiF4 and beta-cyclodextrin inclusion complex and its in vitro evaluation on inhibiting enamel demineralization. Arch Oral Biol.

[11] Gu Z, Li J, Söremark R (1996). Influence of tooth surface conditions on enamel fluoride uptake after topical application of TiF4 in vitro. Acta Odontol Scand.

[12] Büyükyilmaz T, Ogaard B, Rølla G (1997). The resistance of titanium tetrafluoride-treated human enamel to strong hydrochloric acid. Eur J Oral Sci.

[13] Bridi EC, Amaral FL, França FM, Turssi CP, Basting RT (2016). Inhibition of demineralization around the enamel-dentin/restoration interface after dentin pretreatment with TiF4 and self-etching adhesive systems. Clin Oral Investig.

[14] Basting RT, Basting RT, Velarde BS, Bridi EC, França FM, Turssi CP, Amaral FL (2017). Titanium tetrafluoride incorporated into a two-step self-etching adhesive system: physico-mechanical characterization and bonding stability. J Mech Behav Biomed Mater.

[15] Langhorst SE, O’Donnell JN, Skrtic D (2009). In vitro remineralization of enamel by polymeric amorphous calcium phosphate composite: quantitative micro radiographic study. Dent Mater.

[16] Liu Y, Zhang L, Niu LN, Yu T, Xu HH, Weir MD, Oates TW, Tay FR, Chen JH (2018). Antibacterial and remineralizing orthodontic adhesive containing quaternary ammonium resin monomer and amorphous calcium phosphate nanoparticles. J Dent.

[17] Montagner AF, Opdam NJ, Ruben JL, Bronkhorst EM, Cenci MS, Huysmans MC (2016). Behavior of failed bonded interfaces under in vitro cariogenic challenge. Dent Mater.

[18] Klocke A, Kahl-Nieke B (2005). Influence of force location in orthodontic shear bond strength testing. Dent Mater.

[19] Melo MA, Wu J, Weir MD, Xu HH (2014). Novel antibacterial orthodontic cement containing quaternary ammonium monomer dimethyl aminododecyl methacrylate. J Dent.

[20] Tranquilin JB, Bridi EC, Amaral FL, França FM, Turssi CP, Basting RT (2016). TiF4 improves microtensile bond strength to dentin when using an adhesive system regardless of primer/bond application timing and method. Clin Oral Investig.

[21] Al-Gerny YA, Ghorab SM, Soliman TA (2019). Bond strength and elemental analysis of oxidized dentin bonded to resin modified glass ionomer based restorative material. J Clin Exp Dent.

[22] Alexandria AK, Nassur C, Nóbrega CBC, Valença AMG, Rosalen PL, Maia LC (2017). In situ effect of titanium tetrafluoride varnish on enamel demineralization. Braz Oral Res.

[23] Wang P, Gao J, Wang D, Snead ML, Li J, Ruan J (2017). Optimizing concentration of titanium tetrafluoride solution for human dentine remineralization. Arch Oral Biol.

[24] Bridi EC, Amaral FLBD, França FMG, Turssi CP, Basting RT (2018). Influence of dentin pretreatment with 2.5% titanium tetrafluoride on inhibiting caries at the tooth-restoration interface in situ. Arch Oral Biol.

[25] de Souza BM, Santi LRP, de Souza SM, Buzalaf MAR (2018). Magalhães AC effect of an experimental mouth rinse containing NaF and TiF4 on tooth erosion and abrasion in situ. J Dent.

[26] Klocke A, Kahl-Nieke B (2005). Influence of cross-head speed in orthodontic bond strength testing. Dent Mater.

[27] Sharma S, Tandon P, Nagar A, Singh GP, Singh A, Chugh VK (2014). A comparison of shear bond strength of orthodontic brackets bonded with four different orthodontic adhesives. J Orthod Sci.

[28] Cheng HY, Chen CH, Li CL, Tsai HH, Chou TH, Wang WN (2011). Wang, bond strength of orthodontic light-cured resin-modified glass ionomer cement. Eur J Orthod.

[29] Wijeyeweera RL, Kleinberg I (1989). Acid-base pH curves in vitro with mixtures of pure cultures of human oral microorganisms. Arch Oral Biol.

[30] Langhorst SE, O'Donnell JN, Skrtic D (2009). In vitro remineralization of enamel by polymeric amorphous calcium phosphate composite: quantitative microradiographic study. Dent Mater.

[31] Chow LC, Takagi S, Shih S (1992). Effect of a two-solution fluoride mouth rinse on remineralization of enamel lesions in vitro. J Dent Res.

[32] Belli R, Kreppel S, Petschelt A, Hornberger H, Boccaccini AR, Lohbauer U (2014). Strengthening of dental adhesives via particle reinforcement. J Mech Behav Biomed Mater.

[33] Rizvi A, Zafar MS, Al-Wasifi Y, Fareed W, Khurshid Z (2016). Role of enamel deminerlization and remineralization on microtensile bond strength of resin composite. Eur J Dent.

[34] Gorton J, Featherstone JD (2003). In vivo inhibition of demineralization around orthodontic brackets. Am J Orthod Dentofacial Orthop.

[35] Pascotto RC, Navarro MF, Capelozza Filho L, Cury JA (2004). In vivo effect of a resin modified glass ionomer cement on enamel demineralization around orthodontic brackets. Am J Orthod Dentofacial Orthop.

[36] Uysal T, Amasyali M, Koyuturk AE, Sagdic D (2009). Efficiency of amorphous calcium phosphate-containing orthodontic composite and resin modified glass ionomer on demineralization evaluated by a new laser fluorescence device. Eur J Dent.

[37] Featherstone JD, ten Cate JM, Shariati M, Arends J (1983). Comparison of artificial caries-like lesions by quantitative microradiography and microhardness profiles. Caries Res.

[38] Alcântara PC, Alexandria AK, Souza IP, Maia LC (2014). In situ effect of titanium tetrafluoride and sodium fluoride on artificially decayed human enamel. Braz Dent J.

[39] Soliman TA, Othman M (2017). Mechanical properties of the new ketac™ universal glass ionomer restorative material: effect of resin coating. EDJ.

[40] Thimmaiah C, Shetty P, Shetty SB, Natarajan S, Thomas NA (2019). Comparative analysis of the remineralization potential of CPP-ACP with fluoride, tri-calcium phosphate and nano hydroxyapatite using SEM/EDX-an in vitro study. J Clin Exp Dent.

[41] Memarpour M, Shafiei F, Rafiee A, Soltani M, Dashti MH (2019). Effect of hydroxyapatite nanoparticles on enamel remineralization and estimation of fissure sealant bond strength to remineralized tooth surfaces: an in vitro study. BMC Oral Health.

[42] Vitiello F, Tosco V, Monterubbianesi R, Orilisi G (2022). Remineralization efficacy of four remineralizing agents on artificial enamel lesions: sem-eds investigation. Materials (Basel).

[43] Orilisi G, Monterubbianesi R, Notarstefano V, Tosco V, Vitiello F, Giuliani G, Putignano A, Orsini G (2021). New insights from raman microspectroscopy and scanning electron microscopy on the microstructure and chemical composition of vestibular and lingual surfaces in permanent and deciduous human teeth. Spectrochim Acta A Mol Biomol Spectrosc.

